# Activation of neuronal endothelin B receptors mediates pressor response through alpha‐1 adrenergic receptors

**DOI:** 10.14814/phy2.13077

**Published:** 2017-02-20

**Authors:** Bryan K. Becker, Joshua S. Speed, Mackenzie Powell, David M. Pollock

**Affiliations:** ^1^ Division of NephrologyDepartment of MedicineCardio‐Renal Physiology and MedicineUniversity of Alabama at BirminghamBirminghamAlabama

**Keywords:** Blood pressure, hypertension, sympathetic nerves

## Abstract

Abnormalities in activity of the endothelin (ET) system have been widely reported in a number of cardiovascular disease states such as hypertension and heart failure. Although the vascular responses to ET are well established, the interaction between ET and other important modulators of blood pressure, such as the sympathetic nervous system, are less understood. Previous reports implicate ET signaling through ET type B (ET_B_) receptors in increasing neuronal activity. Therefore, we hypothesized that activation of ET_B_ receptors on sympathetic nerves would increase blood pressure through an adrenergic‐mediated mechanism. Thus, we used anesthetized ET_B_‐deficient rats, which only express functional ET_B_ receptors on adrenergic neurons, and genetic controls, which express functional ET_B_ receptors in vascular tissue and kidney epithelium. We determined the pressor response to the selective ET_B_ receptor agonist sarafotoxin c (S6c). Separate groups of rats were treated with the *α*_1_‐adrenergic receptor antagonist prazosin or the *β*‐adrenergic receptor antagonist propranolol to elucidate the role of adrenergic signaling in mediating the blood pressure response. We observed a dose‐dependent pressor response to S6c in ET_B_‐deficient rats that was reversed by prazosin treatment and augmented by propranolol. In genetic control rats, the effects of S6c on sympathetic neurons were mostly masked by the direct activity of ET_B_ receptor activation on the vasculature. Heart rate was mostly unaffected by S6c across all groups and treatments. These results suggest that ET_B_ activation on sympathetic neurons causes an increase in blood pressure mediated through *α*_1_‐adrenergic receptor signaling.

## Introduction

The endothelin (ET) class of peptides has been described as comprising the most potent vasoactive peptides known. Aberrations in ET signaling have been correlated with numerous cardiovascular diseases such as hypertension (Pollock 2000; Speed and Pollock 2013, 2015), heart failure (Schneider et al. 2007; Zucker et al. 2012), and pulmonary hypertension (Kedzierski and Yanagisawa 2001). The physiological actions of ET in mammals occur through the activation of either ET type A (ET_A_) or type B (ET_B_) receptors. These receptors are present on many tissues including endothelium, vascular smooth muscle cells, neurons, and various renal tissues. Acute intravenous infusion of ET‐1 causes a transient reduction in blood pressure followed by a prolonged increase in pressure (Kohan et al. 2011). It is typically thought that this phenomenon is due to the initial binding of ET‐1 to ET_B_ on vascular endothelium, which results in NO‐mediated vascular dilation and a resultant drop in blood pressure. This is immediately followed by activation of ET_A_ and, to some extent, ET_B_ on smooth muscle cells, which causes sustained smooth muscle contraction and increases blood pressure. Although this vascular phenomenon of ET signaling has been relatively well studied, less is known about the effects of ET on other systems important in blood pressure control such as the sympathetic nervous system.

Sympathetic neuronal activity has many effects on blood pressure both in relation to long‐term control of blood pressure and short‐term modulation of vascular tone.With regard to the sympathetic control of vascular tone, norepinephrine, the primary effector neurotransmitter of postganglionic sympathetic neurons binding to *α*_1_‐adrenergic receptors on smooth muscle results in contraction, increased vascular resistance, and increased blood pressure. Many factors influence sympathetic control of blood pressure such as stress and angiotensin signaling; however, not much is known about how ET interacts with sympathetic neurons to modulate blood pressure despite the clear evidence of receptor expression within the sympathetic nerves (Takimoto et al. 1993; Rubanyi and Polokoff 1994). There is some indication that ET_B_ receptors on sympathetic neurons can increase neuronal activity (Dai et al. 2004; D'Angelo et al. 2005, 2006; Li et al. 2008; Kopp et al. 2009), yet direct evidence connecting ET_B_ receptors and sympathetic activity in modulating blood pressure is lacking. ET signaling may play an important role in acute control of blood pressure during sympathetic activation during instances such as the response to stressful stimuli (Wilbert‐Lampen et al. 2010); however, the mechanisms by which ET interacts with sympathetic control of acute changes in blood pressure is not well elucidated. We hypothesized that activation of ET_B_ receptors on sympathetic nerves would increase blood pressure through an adrenergic‐mediated mechanism.

To investigate our hypothesis, we infused the selective ET_B_ receptor agonist (Davenport et al. 2016) sarafotoxin c (S6c) in ET_B_‐deficient (ET_B_‐def) rats that lack functional ET_B_ receptors except on adrenergic tissues and littermate controls (Gariepy et al. 1998). We also treated rats with either the *α*_1_‐adrenergic receptor blocker prazosin or the *ß*‐adrenergic receptor blocker propranolol to determine the contribution of the adrenergic system in ET activation of sympathetic control. Because of the well‐established receptor selectivity of S6c (Williams et al. 1991; Sokolovsky 1992), the use of the selective agonist instead of ET‐1 will limit effects to ET_B_ receptor signaling, and, in the case of the ET_B_‐def rats, this effect was further isolated to adrenergic sympathetic neurons as these are the only tissues expressing functional ET_B_ receptors in this strain of rat.

## Materials and Methods

### Animal use

A total of 27 male control and ET_B_‐def rats were used in the following experiments. Rats were aged 10–14 weeks old and weighed 300–350 g at time of the experimental protocol from a colony housed on site. All procedures were approved by the Institutional Animal Care and Use Committee of the University of Birmingham at Alabama and were conducted in accordance with the Guide for the Care and Use of Laboratory Animals of the NIH.

Our animals are originally derived from the spotting‐lethal rat that carries a natural 301‐bp deletion in the ET_B_ receptor that lacks expression of functional ET_B_ receptors. Rats homozygous for this mutation display a lethal phenotype of congenital intestinal megacolon and are commonly used as a model for Hirschsprung's disease (Gariepy et al. 1996). Gariepy et al. (1998) used a dopamine‐*ß*‐hydroxylase promoter to direct the transgenic (TG) ET_B_ receptor expression in adrenergic tissue that “rescued” the rats from the intestinal defect. The resulting ET_B_ def express functional ET_B_ receptors only in nervous tissue while transgenic littermate controls (TG controls) express the transgene as well as normal ET_B_ receptor expression in vascular and other tissues.

### Experimental protocol

Animals were prepared in a manner similar to previously published studies (Pollock et al. 2000; Speed et al. 2015). In brief, all rats were anesthetized by an i.p. injection of 100 mg/kg of Inactin (thiobutabarbital sodium salt hydrate; Sigma‐Aldrich, St. Louis, MO), and extent of anesthesia was evaluated by the absence of reflexive withdraw to painful stimuli such as foot pinch. The trachea was canulated with P‐10 tubing to facilitate free breathing and the carotid artery and external jugular vein were catheterized with PE‐50 tubing for measurement of arterial pressure and IV infusion of agents, respectively. Arterial blood pressure was measured via a pressure transducer and collected on LabChart (AD Instruments, Colorado Springs, CO). Animals were allowed to recover for 30 min following instrumentation to stabilize baseline hemodynamic parameters.

Following stabilization, animals were given an IV infusion of 5 mg/kg chlorisondamine (Sigma‐Aldrich, St. Louis, MO). After 10 min, an IV infusion of vehicle (10% ethyl alcohol in saline), 1 mg/kg prazosin, or 5 mg/kg propranolol was administered. Following an additional 10 min recovery period, S6c (American Peptide, Fisher Scientific, Sunnyvale, CA) was given in a 1–2 sec bolus in concentrations of 0.1, 0.3, and 1 nmol/kg with each infusion separated by a period of 15 min. The order of administered doses was the same in all animals.

### Statistics

Responses to S6c were compared by calculating the area under the curve. Pulse pressure (PP) is displayed as percent change from baseline because slight variations in catheter placement between subjects influenced absolute values. All data are expressed as mean ± standard error of the mean. Comparisons between groups were analyzed by an unpaired *t*‐test or two‐way analysis of variance (ANOVA) followed by a Tukey's post hoc test for multiple comparisons; *P* values of less than 0.05 were considered statistically significant.

## Results

### Baseline characteristics

**Figure 1:**
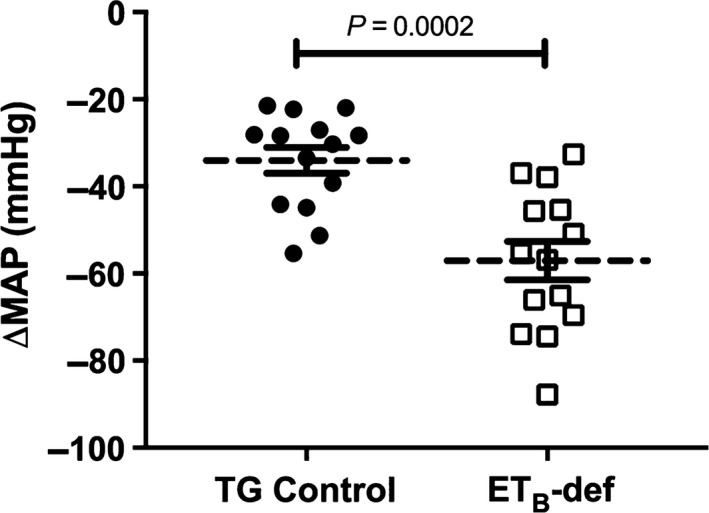
Effect of chlorisondamine on mean arterial pressure in transgenic (TG) control and endothelin type B‐deficient (ET_B_‐def) rats. Individual responses relative to pre‐chlorisondamine baseline are plotted with mean ± SEM. Unpaired *t*‐test.

**Figure 2:**
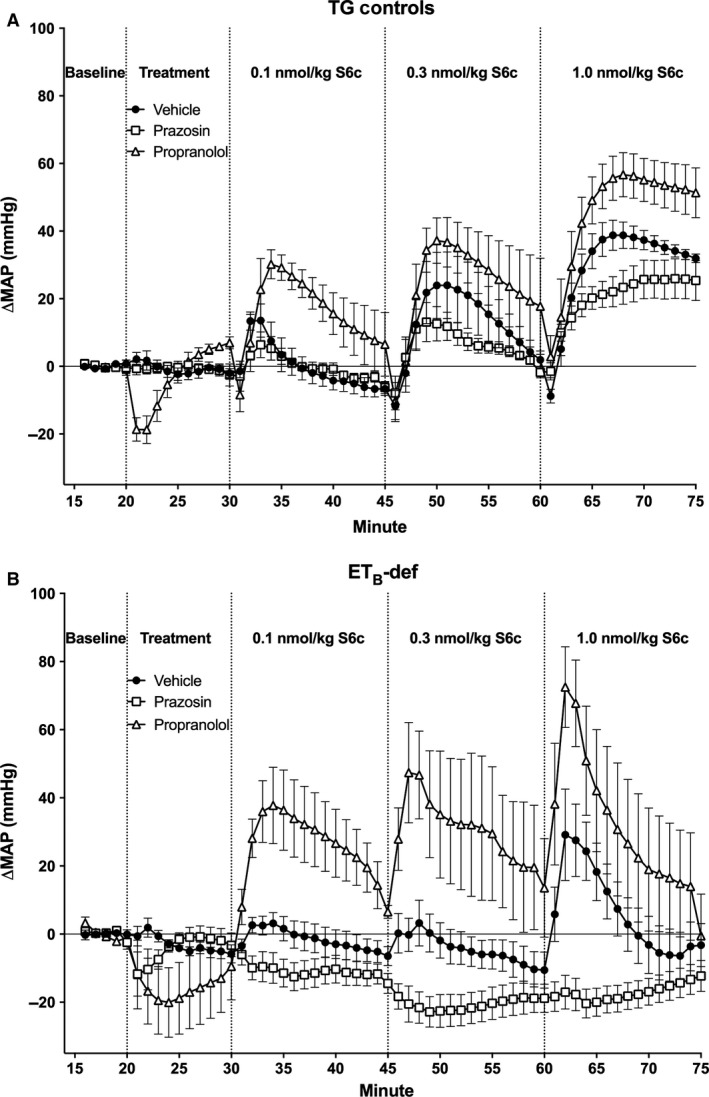
Minute averages of changes in MAP to infusion of S6c in TG control (A) and ET_B_‐def (B) rats treated with vehicle, prazosin, or propranolol. TG Vehicle *n* = 4; TG Prazosin *n* = 5; TG Propranolol *n* = 4; ET_B_‐def Vehicle *n* = 5; ET_B_‐def Prazosin *n* = 5; ET_B_‐def Propranolol *n* = 4. MAP, mean arterial pressure; S6c, sarafotoxin c; TG, transgenic; ET_B_‐def, endothelin type B‐deficient.

Similar to previously published studies, we observed a higher resting mean arterial pressure (MAP) in ET_B_‐def rats compared to TG controls (121.30 ± 5.82 vs. 91.75 ± 3.69 mmHg; *n* = 13–14/group; *P* = 0.0003). There was a larger peak depressor response to chlorisondamine in ET_B_‐def rats compared to TG controls (Fig. 1). ET_B_‐def and TG control rats exhibited similar hemodynamic characteristics following ganglionic blockade (MAP = 88.34 ± 2.85 vs. 80.02 ± 2.90 mmHg, respectively; *P* = 0.052) (heart rate, HR = 331.4 ± 12.44 vs. 338.5 ± 7.431 bpm, respectively; *P* = 0.63) (PP = 37.6 ± 5.0 vs. 43.1 ± 3.8 mmHg, respectively; *P* = 0.4). Treatment with vehicle or prazosin did not significantly affect MAP, HR, or PP (Figs. 2, 4, 6, 4, and 6). Propranolol transiently decreased MAP and HR and increased PP, all of which returned to near pre‐treatment, baseline levels before the administration of S6c (Figs. 2, 4, 6, 4, and 6).

### Responses to S6c

**Figure 3:**
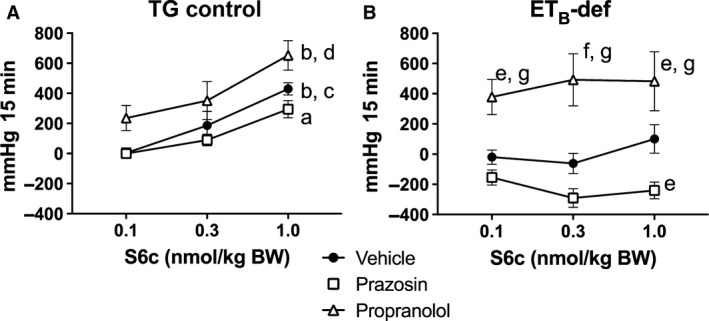
Quantification of changes to mean arterial pressure following S6c in TG control (A) and ETB‐def (B) rats expressed as area under the curve. ^a^*P* < 0.01, ^b^*P* < 0.001 versus 0.1 nmol/kg S6c; ^c^*P* < 0.05, ^d^*P* < 0.01 versus 0.3 nmol/kg S6c; ^e^*P* < 0.05, ^f^*P* < 0.001 versus vehicle; ^g^*P* < 0.001 versus Prazosin; TG Vehicle *n *=* *4; TG Prazosin *n *=* *5; TG Propranolol *n *=* *4; ETB‐def Vehicle *n *=* *5; ETB‐def Prazosin *n *=* *5; ETB‐def Propranolol *n *=* *4; two‐way ANOVA. BW, body weight; S6c, sarafotoxin c; TG, transgenic; ETB‐def, endothelin type B‐deficient; ANOVA, analysis of variance.

Mean arterial pressure responded to S6c in the classic biphasic and dose‐dependent manner in TG control rats (Figs. 2A and 3A). There was a rapid and transient depressor effect followed by a robust and sustained increase in pressure (Fig. 2A). Although adrenergic blockade with propranolol tended to augment the pressor response, and prazosin tended to attenuate the pressor response to S6c, these differences did not reach significance in TG control rats (Fig. 3A). In contrast, ET_B_‐def rats lacked a dose‐dependent response to S6c as measured by area under the curve, but did have a strong treatment effect following adrenergic blockade (Figs. 2B and 3B). Vehicle treatment resulted in a small pressor response to S6c, but as expected, completely lacked the rapid, transient depressor effect seen in the TG control group. The pressor response to S6c in ET_B_‐def rats was greatly augmented by antagonism of *ß*‐adrenergic receptors relative to vehicle, and inhibition of *α*_1_‐adrenergic receptors with prazosin resulted in a depressor response to S6c (Fig. 3B).

**Figure 4:**
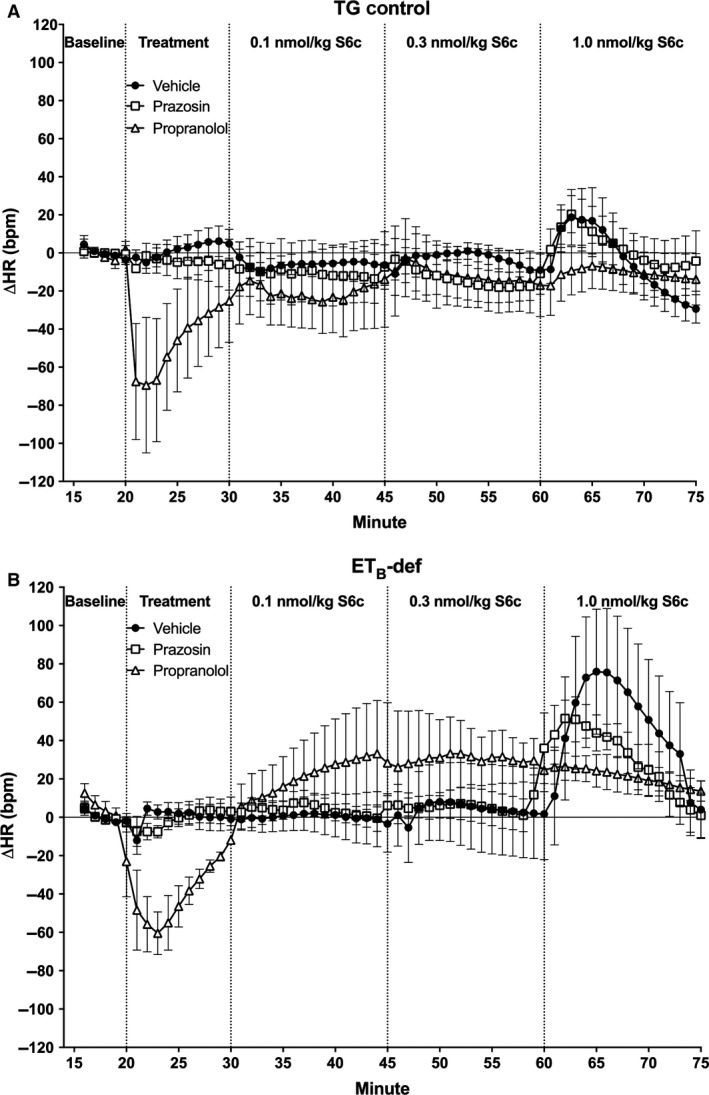
Minute averages of changes in (HR) to infusion of S6c in TG control (A) and ET_B_‐def (B) rats treated with vehicle, prazosin, or propranolol. TG Vehicle *n* = 4; TG Prazosin *n* = 5; TG Propranolol *n* = 4; ET_B_‐def Vehicle *n* = 5; ET_B_‐def Prazosin *n* = 5; ET_B_‐def Propranolol *n* = 4. HR, heart rate; S6c, sarafotoxin c; TG, transgenic; ET_B_‐def, endothelin type B‐deficient.

**Figure 5:**
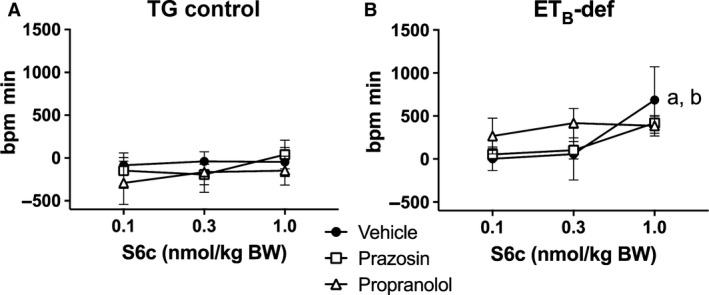
Quantification of changes to heart rate following S6c in TG control (A) and ET_B_‐def (B) rats expressed as area under the curve. ^a^ *P *<* *0.05 versus 0.1 nmol/kg S6c; ^b^ *P *<* *0.05 versus 0.3 nmol/kg S6c; TG Vehicle *n* = 4; TG Prazosin *n* = 5; TG Propranolol *n* = 4; ET_B_‐def Vehicle *n* = 5; ET_B_‐def Prazosin *n* = 5; ET_B_‐def Propranolol *n* = 4.; two‐way ANOVA. BW, body weight; S6c, sarafotoxin c; TG, transgenic; ET_B_‐def, endothelin type B‐deficient; ANOVA, analysis of variance.

HR was mostly unaffected by S6c with the only significant response being a tachycardia that occurred in the ET_B_‐def, vehicle treated animals following the highest dose of S6c (Figs. 4, 5).

**Figure 6:**
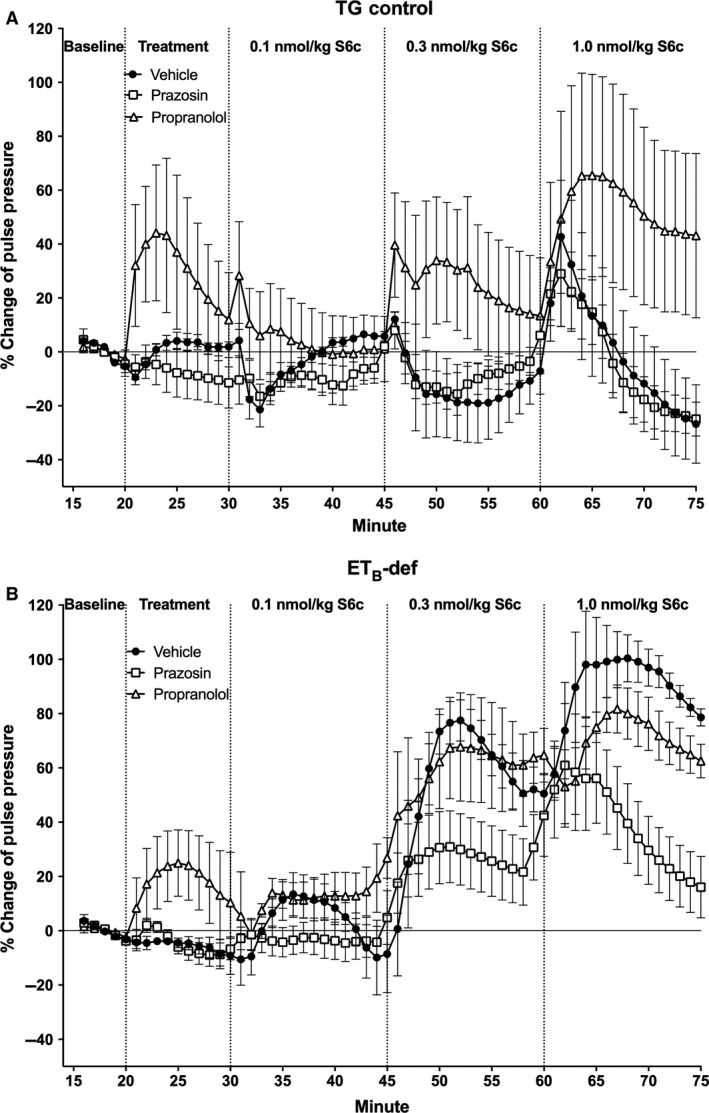
Minute averages of changes in pulse pressure as expressed as percent change of baseline to infusion of S6c in TG control (A) and ET_B_‐def (B) rats treated with vehicle, prazosin, or propranolol. TG Vehicle *n* = 4; TG Prazosin *n* = 5; TG Propranolol *n* = 4; ET_B_‐def Vehicle *n* = 5; ET_B_‐def Prazosin *n* = 5; ET_B_‐def Propranolol *n* = 4. S6c, sarafotoxin c; TG, transgenic; ET_B_‐def, endothelin type B‐deficient.

**Figure 7:**
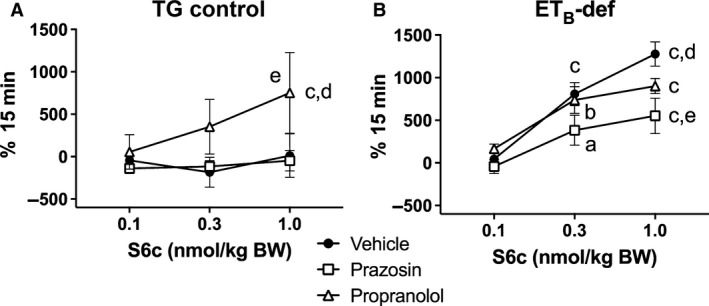
Quantification of changes to pulse pressure following S6c in TG control (A) and ET_B_‐def (B) rats expressed as area under the curve. ^a^ *P* < 0.05, ^b^ *P* < 0.01, ^c^ *P *< 0.001 versus 0.1 nmol/kg S6c; ^d^ *P* < 0.05 versus 0.3 nmol/kg S6c; ^e^ *P* < 0.05 versus prazosin; TG Vehicle *n* = 4; TG Prazosin *n* = 5; TG Propranolol *n* = 4; ET_B_‐def Vehicle *n* = 5; ET_B_‐def Prazosin *n* = 5; ET_B_‐def Propranolol *n* = 4.; two‐way ANOVA. BW, body weight; S6c, sarafotoxin c; TG, transgenic; ET_B_‐def, endothelin type B‐deficient; ANOVA, analysis of variance.

Pulse pressure in the TG control strain was only affected by S6c after treatment with propranolol in which PP increased in a dose‐dependent manner and was significantly higher than vehicle and prazosin treatment (Figs. 6A and 7A). Regardless of treatment, PP increased in a dose‐dependent manner in ET_B_‐def rats, and prazosin significantly attenuated the increase in PP after the highest dose of S6c (Figs. 6B and 7B).

## Discussion

The major finding of this study is that activation of ET_B_ receptors on adrenergic tissue causes increased blood pressure mediated through *α*_1_‐adrenergic receptor signaling in ganglion‐blocked rats. An additional finding is that ET_B_‐def rats have elevated sympathetic tone as evidenced by the greater reduction in MAP following ganglionic blockade compared to TG control rats.

Direct vascular responses to ET‐1 have been relatively well characterized. In the classical vascular reaction to exogenously applied ET‐1, initial binding of ET‐1 to ET_B_ receptors on vascular endothelial cells elicits production of nitric oxide and prostacyclins, which in turn mediate smooth muscle relaxation causing a transient reduction in vascular resistance and blood pressure (Berti et al. 1993; Filep et al. 1993a,b). Evidence for this vasodilatory effect of ET_B_ receptors has also been demonstrated by infusion of ET‐1 in ET_B_‐def rats, which lack endothelial ET_B_ receptors, and results in a vasoconstriction without the initial vasodilatory response (Pollock et al. 2000). The present study further confirms this mechanism (Fig. 2). Following the transient vasodilation, ET‐1 binding to both ET_A_ and ET_B_ receptors on the vascular smooth muscle result in a potent vasoconstriction mediated by common pathways involving phospholipase C/IP3‐mediated Ca^2+^ influx and activation of the Ras/Raf/ERK pathway (Rubanyi and Polokoff 1994; Molero et al. 2002; Pollock et al. 2005; Chen et al. 2009). In summary, both ET_A_ and ET_B_ receptors on smooth muscle contribute to the sustained pressor response to ET‐1, whereas ET_B_ receptors on endothelium are known to contribute to the initial, transient depressor action of ET‐1. Here we demonstrate that in addition to these previously described direct vascular actions of ET_B_ receptors, activation of sympathetic neurons by ET_B_ receptors contributes to increased blood pressure mediated by activation of *α*_1_‐adrenergic receptors.

Isolating the effects of ET‐1 to autonomic neuronal control of blood pressure has been difficult in vivo because of the potent vascular effects of ET‐1; however, certain animal models, such as the ET_B_‐def rat, are useful in evaluating the specific actions of ET‐1 on neurons. In previous studies, the ET_B_ receptor agonist S6c was infused into ET_B_‐def and TG control animals (Pollock et al. 2000). The control animals displayed the traditional, yet blunted, ET‐1 response to the ET_B_ receptor agonist, a transient depressor/vasodilation followed by a more sustained vasoconstriction and increase in blood pressure. The ET_B_‐def rats lacked the vasodilation because they lack functional ET_B_ receptors on endothelium; however, they retained the sustained vasoconstriction response. Because these rats only have functional ET_B_ receptors on adrenergic tissue, it stands to reason that the pressor response to ET_B_ agonism is mediated predominantly by sympathetic activation.

Previous studies have reported an increase in peripheral sympathetic neuron reactive oxygen species generation following ET_B_ receptor activation (Dai et al. 2004; Lau et al. 2006), which increases neuronal activity. Additionally, ET has been implicated in regulation of cardiac sympathetic tone (Reid et al. 1989; Kumar et al. 1997; Yamamoto et al. 2004; Isaka et al. 2007; Abukar et al. 2016). In a series of experiments using anesthetized dogs, Morimoto's group described an inhibitory effect of intrarenal infusions of S6c on renal nerve stimulation‐induced antidiuresis and norepinephrine overflow suggesting an inhibitory role for ET_B_ receptors on efferent sympathetic nerve terminal release of norepinephrine (Matsumura et al. 1996; Matsuo et al. 1997a,b). In contrast to these findings, ET_B_ receptor activity has been shown to increase norepinephrine release from cardiac sympathetic nerves (Isaka et al. 2007). This apparent conflict may suggest differing actions of ET receptors among various sympathetic beds or even differing actions of ET receptors within neurons such that presynaptic and postsynaptic ET receptor binding may exert divergent effects on activity and neurotransmitter release. Our data suggest that on vascular sympathetic beds, activation of neuronal ET_B_ receptors increases norepinephrine release resulting in *α*_1_‐adrenergic receptor mediated increases in blood pressure. Related to this finding are previous results demonstrating a selective activation of sympathetic vasomotor tone to the splanchnic vascular bed indicating differential sympathetic activation by S6c (Li et al. 2008). The absence of a change in HR suggests that postganglionic cardiac sympathetic neurons in our model may not be influenced by ET_B_ receptor activation particularly at low doses of S6c.

In the present study, we sought to isolate the effect of ET_B_ receptor activation on neuronal control of blood pressure in vivo by two methods: (1) the use of the ET_B_‐def rat, which only expresses ET_B_ receptors in adrenergic tissue; (2) the use of a selective ET_B_ receptor agonist, S6c. Infusion of S6c in TG control rats exhibited the classical vascular response with the transient, endothelium‐dependent vasorelaxation followed by a sustained increase in pressure. These data confirm previous reports and provide further evidence in support of the sustained vasoconstriction response to ET_B_ receptor activation on smooth muscle (Pollock et al. 2000; Speed et al. 2015). Conversely, ET_B_‐def rats, which lack functional ET_B_ receptors on endothelium and smooth muscle, only exhibit a pressor response to S6c when mediated through activation of sympathetic nerves and *α*_1_‐adrenergic signaling. Furthermore, the response to S6c was qualitatively blunted in ET_B_‐def rats compared to controls likely due to the lack of vascular responses to ET_B_ receptor activation. Evidence in support of this conclusion is that the pressor response was abolished by *α*_1_‐adrenergic receptor blockade with prazosin (Figs. 2, 3). We also conclude that this sympathoexcitatory effect of ET_B_ agonism was through direct activation of peripheral, postganglionic vasomotor sympathetic nerves because ganglionic blockade by chlorisondamine would preclude the involvement of higher‐order neurons in this effect. Prazosin did not abolish the pressor response to S6c in TG‐controls, but this is expected as the contribution of sympathetic activation from S6c is mostly overcome by its direct vascular effects. However, even within the TG‐control groups, the effects of sympathetic activation are present as prazosin tended to attenuate and propranolol tended to augment the response to S6c.

Our results clarify previous reports from our laboratory suggesting a role for ET_B_ receptors in enhancing sympathetic nerve activity‐mediated increases in blood pressure following acute stress (D'Angelo et al. 2005, 2006). The first of these studies demonstrated an augmented pressor response to acute air jet stress following ET_A_ receptor antagonism that was attenuated following ET_B_ receptor antagonism in Sprague‐Dawley rats (D'Angelo et al. 2005). These findings suggested a “non‐classical” role for ET receptors in sympathetic neurons as compared to the vasculature, namely that ET_B_ receptors are involved in sympathetic activation increasing blood pressure, and ET_A_ receptors are involved in blunting sympathetic activity. These findings were further explored in the Dahl salt sensitive model of hypertension. Similar to what was demonstrated in the initial study, ET_A_ receptor antagonism augmented the pressor response to acute air jet stress (D'Angelo et al. 2006). Our results provide further, specific evidence that ET_B_ receptors on sympathetic neurons increase adrenergic activity. Additionally, our results also suggest that postganglionic sympathetic nerves are activated by ET_B_ receptors as we performed our experiments under pharmacological ganglionic blockade.

The effects of S6c on PP are also consistent with an ET_B_‐dependent sympathetic‐mediated vasoconstriction. PP is inversely related to aortic compliance and directly related to ventricular stroke volume. There were minimal effects of S6c on HR in all animals (Figs. 4, 5), but as we did not directly evaluate ventricular hemodynamics in the current study, we are unable to conclusively exclude effects on stroke volume. Arterial compliance is a likely target for the differences observed in relation to PP. In TG controls, infusion of S6c only had an effect on increasing PP in the presence of propranolol, and vehicle and prazosin treatment groups did not respond to S6c. This suggests that the adrenergic‐mediated effects of S6c on vascular tone in TG rats was only unmasked when pharmacological blockade isolated the influence of *α*_1_‐adrenergic receptors. Similarly, in ET_B_‐def rats, prazosin blunted the increase in PP following S6c suggesting an *α*_1_‐adrenergic‐mediated vascular constriction to neuronal ET_B_ receptor activation.

Another finding of the present study is the observation that ET_B_‐def rats have elevated basal sympathetic tone as evidence by the greater response to ganglionic blockade. It is unclear from the present study what is directly responsible for this higher sympathetic activity although a few possibilities exist. Bruno et al. (2011) demonstrated a sympathoexcitatory effect of ET‐1 in both normotensive and hypertensive patients, the sensitivity of which was higher in hypertensive subjects. The ET_B_‐def rat has previously been reported to have high circulating plasma concentrations of ET‐1 presumably due to the lack of ET_B_ receptor‐mediated clearance mechanisms. It is possible that the increased circulating levels of ET‐1 mediate an increase in sympathetic tone by direct activation of postganglionic nerurons, although the physiological role of circulating ET‐1 is thought to play a minor role compared to its autocrine and paracrine functions. Although not previously measured, the elevated plasma levels of ET‐1 likely lead to higher concentrations in cerebrospinal fluid. Neurons involved in autonomic control of blood pressure throughout the central and peripheral nervous systems express ET_A_ and ET_B_ receptors. Elegant autoradiographic binding studies in rats have shown that labeled ET‐1 introduced intravenously binds brain areas lacking an intact blood–brain barrier such as the subfornical organ (Koseki et al. 1989), which has been widely implicated in mediating sympathoexcitation in numerous forms of hypertension (Osborn et al. 2012; Wei et al. 2013; Saxena et al. 2015; Collister et al. 2016). When brain sections are incubated in autoradiographic ET‐1, nearly the entire section displays robust labeling indicating promiscuous neuronal binding of ET‐1, at least within the central nervous system (Koseki et al. 1989). Intracerebroventricular application of ET‐1 increases arterial pressure (Nishimura et al. 1990, 1991; Yamamoto et al. 1992; Stocker et al. 2001) and vasopressin release (Yamamoto et al. 1992; Matsumura et al. 1994), but it is unclear if these effects are due to the direct actions of ET‐1 on neurons or secondary to vascular effects. Furthermore, even less is known about the direct actions of ET‐1 on peripheral sympathetic neurons. This could have a direct effect on activating presympathetic neurons in cardiovascular control areas, such as the subfornical organ. Furthermore, this suggests but does not directly address the question if this sympathetic tone contributes to the elevated blood pressure previously observed in this strain (D'Angelo et al. 2005; Sullivan et al. 2006; Speed et al. 2015). Further study is warranted to evaluate the contribution of the autonomic nervous system to the hypertensive phenotype in ET_B_‐def rats.

One potential limitation of this study is that all experiments were performed under anesthesia. We chose Inactin as the anesthetic due to its wide use in autonomic studies and its minimal impact on autonomic function; however, we cannot exclude the potential influences of anesthesia on neuronal function in our preparation as the use of any anesthesia introduces potential limitations. Furthermore, there are suggestions that Inactin itself may alter adrenergic signaling (Tucker et al. 1982). Choice of anesthesia is also less likely of a factor in our experimental design because all animals were given ganglionic blockade, thus the hemodynamic consequences of infusion of S6c are likely only due to postganglionic action removing the influence of central control of blood pressure. This, however, also presents an additional limitation in our ability to apply our findings to central and preganglionic neurons. Our results suggest a direct contribution of ET_B_ receptors in sympathetic activation and increases in blood pressure on peripheral adrenergic nerves, but we are unable to comment directly on the function of ET_B_ receptors in other neuronal populations. Further study is warranted investigating the contribution of the ET system toward neuronal control of hemodynamic function. Another limitation is the acute nature of our experimental approach, which limits its direct translational ability toward understanding more chronic forms of cardiovascular control.

In summary, our results demonstrate that ET_B_ receptor activation increases sympathetic neuronal activity and increases blood pressure through an *α*_1_‐adrenergic mechanism. In rats deficient of ET_B_ receptors except on adrenergic tissue, blockade of *α*_1_‐adrenergic receptors completely prevented the pressor response to ET_B_ receptor activation and blockade of *β*‐adrenergic receptors augmented this response. The influence of ET_B_ receptor activation of adrenergic‐mediated increases in pressure was present in TG control rats although minimized due to the direct vascular effects of ET_B_ receptor activation. We also observed an increased basal sympathetic tone in rats deficient of functional ET_B_ receptors. These results provide evidence in support of a pro‐sympathetic action of ET_B_ receptors on sympathetic neurons followed by an *α*_1_‐adrenergic‐mediated pressor response.

## Conflict of Interest

No conflicts of interest, financial or otherwise, are declared by the author(s). M. P. contributed to this work as a part of the University of Alabama at Birmingham SeCURE program, Summer Cardio‐renal Undergraduate Research Experience.
